# Treatment of Stress Urinary Incontinence by Cinnamaldehyde, the Major Constituent of the Chinese Medicinal Herb *Ramulus Cinnamomi*

**DOI:** 10.1155/2014/280204

**Published:** 2014-03-10

**Authors:** Yung-Hsiang Chen, Yu-Ning Lin, Wen-Chi Chen, Wen-Tsong Hsieh, Huey-Yi Chen

**Affiliations:** ^1^Department of Pharmacology, Graduate Institute of Integrated Medicine, College of Chinese Medicine, China Medical University, Taichung 40402, Taiwan; ^2^Departments of Medical Research, Urology, and Obstetrics and Gynecology, China Medical University Hospital, Taichung 40402, Taiwan

## Abstract

Stress urinary incontinence (SUI) is a common disorder in middle-aged women and the elderly population. Although surgical treatment of SUI has progressed, pharmacological therapies remain unelucidated. We screened potential herbal medicines against SUI with an *ex vivo* organ bath assay. *Ramulus Cinnamomi* and its major constituent cinnamaldehyde cause a high contractile force of the urethra and a low contractile force of blood vessels. Cinnamaldehyde dose-dependently reduced lipopolysaccharide-induced nitric oxide (NO) production and inducible nitric oxide synthase (iNOS) expression in RAW 264.7 cells. In the vaginal distension- (VD-) induced SUI model in mice, cinnamaldehyde significantly reversed the VD-induced SUI physical signs and reduced blood pressure. Cinnamaldehyde may offer therapeutic potential against SUI without the possible side effect of hypertension. The modulation of several SUI-related proteins including myosin, iNOS, survival motor neuron (SMN) protein, and superoxide dismutase 3 (SOD3) may play some crucial roles in the therapeutic approach against SUI. This information may offer clues to the pathogenesis of SUI and open additional avenues for potential therapy strategies.

## 1. Introduction

Stress urinary incontinence (SUI) is a common disorder in recent decades, given the steep increases in the elderly population [1, 2]. It is also a common medical condition affecting middle-aged women worldwide. Approximately 40% of younger women (20–45 years old) suffer from SUI that significantly affects their quality of life [3]. Although progress has been made in the surgical treatment of SUI, pharmacological therapy for SUI is poorly understood. Therefore, drug discovery and development for SUI is urgent. An inconclusive therapy for SUI, which causes urethral contractions, includes *α*-adrenergic agonists such as ephedrine, phenylephrine (PE), midodrine, norfenefrine, phenylpropanolamine, and imipramine. Treatment of women with SUI using *α*-adrenergic stimulating drugs was withdrawn because of unwanted side effects including insomnia, restlessness, elevated blood pressure, arrhythmia, chest pain, and headache [4].

Since conventional therapy is not completely efficacious and older individuals may be unwilling to undergo surgical treatment, alternative treatments may be potentially used as an adjunctive therapy for SUI episodes [5]. Reported traditional Chinese herbal medicines for SUI include several herbs and polyherbal formulae, but evidence regarding the benefits is often lacking. Recently, integrative medicine has been commonly used for health conditions in middle-aged women, and several herbal therapies have been tested to determine if they ameliorate the physical signs of SUI or not. In an uncontrolled trial, subjects were given a Chinese herbal formula; 78% of them experienced decreased frequency of incontinent episodes [6], suggesting the potential application of Chinese herbal medicine for SUI treatment [7–9].

Comprehending the urethral function and pharmacology may lead to the development of promising new agents [10], which may be useful in the management of SUI in women [4]. Because of concerns about *α*-adrenergic agonists, both practitioners and patients are seeking alternatives to clinical *α*-adrenergic stimulating drugs for managing SUI physical sign. In the present study, several candidate herbal medicines were screened and botanical agents for SUI were proposed for development. This study established an* ex vivo* organ bath,* in vitro* cell culture, and* in vivo* animal SUI models to evaluate potential botanical agents against SUI and to explore the possible mechanisms. Among these botanical herbs,* Ramulus Cinnamomi* (the dry twig of* Cinnamomum cassia* Presl, a plant in the family Lauraceae) and its major constituent cinnamaldehyde caused a high contractile force of the urethra and a low contractile force of blood vessels. Plant-derived essential oils have long been used as flavoring agents in agricultural food and beverages and as natural agents for food preservation.* Cinnamomum cassia* bark oil, known as cinnamon oil, is commonly used in the food industry because of its special aroma. Cinnamaldehyde is commonly used in agricultural food and beverages and with great commercial and possible medical value [11]. These analyses will give an insight of pharmacologic treatment of SUI. This information may offer clues to the pathogenesis of SUI and open additional avenues for potential therapy strategies.

## 2. Materials and Methods

### 2.1. Organ Bath Experiment for Potential SUI Drug Screening

Organ bath experiment was used for functional evaluation of contractile urethra and blood vessel* ex vivo* for the contractility study [12, 13]; female pig urethra and blood vessel were prepared from the basal part of the bladder body and renal artery, respectively [13]. Urethra and renal artery were mounted between platinum-plate electrodes and secured by small clips in a double-jacketed organ bath containing 20 mL Krebs' solution aerated with 95% O_2_ and 5% CO_2_ to obtain a pH of 7.4 at 37°C. The composition of the Krebs' solution was (mM): 133 NaCl, 4.7 KCl, 2.5 CaCl_2_, 16.3 NaHCO_3_, 1.35 NaH_2_PO_4_, 0.6 MgSO_4_, and 7.8 dextrose. Isometric contraction was recorded with a computerized data acquisition program (Biobench, National Instruments Corporation, TX, USA) at a rate of 50 Hz and stored on a hard drive for later analyses. At the end of the experiment, the length and weight of each muscle strip between the suspension clips were measured. The *α*-adrenergic agonist PE was used as a positive control.

### 2.2. Cell Culture

Raw 264.7 cells were purchased as cryopreserved cultures from Bioresource Collection and Research Center (Hsinchu, Taiwan). 1 × 10^6^ cells were seeded into 100 mm Petri dishes and incubated at 37°C in 90% Dulbecco's modified Eagle's medium with 4 mM L-glutamine adjusted to contain 1.5 g/L sodium bicarbonate and 4.5 g/L glucose + 10% fetal bovine serum. Primary human bladder smooth muscle cells (HBdSMC) (ScienCell, CA, USA) were grown in the smooth muscle cell medium (SMCM, Catalog number 1101). By which time the cells had reached 90% confluence, the cells were plated at 96-well or 10 cm plates for MTT assay or Western blot [14, 15], respectively.

### 2.3. MTT Assay for Cell Viability

Cells were grown in 96-well plates at a concentration of 3 × 10^4^ cells/well for 24 h, and then serum-free medium with different drugs was added. The cells were collected after 24 h later. Mitochondrial dehydrogenase activity was measured as an index of cell viability using the 3-(4,5-dimethylthiazol-2-yl)-2,5-diphenyl tetrazolium bromide (MTT) assays [16]. In brief, MTT (0.5 mg/mL) was applied to the cells for 4 h to allow the conversion of MTT into formazan crystals, the cells were lysed with dimethyl sulfoxide (DMSO), and the absorbance was read at 570 and 650 nm with a DIAS Microplate Reader (Molecular Devices, CA, USA). The reduction in optical density caused by treatment was used as a measurement of cell viability, normalized to the cells incubated in control medium, which were considered 100% viable.

### 2.4. Nitric Oxide Assay

Cells were grown in 96-well plates at a concentration of 3 × 10^4^ cells/well for 24 h, and then serum-free medium with different drugs was added. Culture supernatants were collected after 24 h later. Nitrite levels were measured by addition of 0.1 mL of the Griess reagent (1.5% sulfanilamide and 0.1% naphthylethylenediamine dihydrochloride in 2.5% H_3_PO_4_) to 0.1 mL of culture supernatant in 96-well plates, leaving the plates in the dark for 10 min, and measuring the color intensity with an automated microtiter plate reader at 550 nm [17].

### 2.5. Experimental Animals and Study Design

Twenty virgin female C57BL/6 strain mice, aged approximately 6–8 weeks, were randomized into 4 groups: a noninstrumented control group; the other mice undergoing vaginal distension (VD) for 1 h with 8 mm dilators (compatible with the diameter of mouse newborn head) were randomized to receive PE (25 *μ*g/kg, i.p.) [18], cinnamaldehyde (150 mg/kg, i.p.) [19], or the vehicle for 3 days. Four days after VD, mice underwent suprapubic bladder tubing (SPT) placement. Six days after VD, leak point pressure (LPP) and maximal urethral closure pressure (MUCP) measurements were assessed in these mice under urethane (1 g/kg, i.p.) anesthesia [20, 21]. Blood pressure (BP) and heart rate (HR) were measured using a BP Monitor for Rats and Mice Model MK-2000 (Muromachi Kikai, Tokyo, Japan) according to the instructions of the manufacturer [22]. After measuring LPP, MUCP, BP, and HR, the animals were sacrificed, and the urethras were removed for Western blot analysis. All experimental protocols were approved by the Institutional Animal Care and Use Committee of China Medical University.

### 2.6. Vaginal Distension

Mice in the VD groups were anesthetized with 1.5% isoflurane. To avoid rupturing the vagina, vaginal accommodation of Hegar's dilators was achieved by sequentially inserting and removing different increasing sizes of Hegar's dilators that were lubricated with Surgilube (Fougera, Melville, NY, USA). Finally, for the VD group, an 8 mm dilator was lubricated and inserted into the vagina [23]. After 1 h, the 8 mm dilator was removed, and the animal was allowed to awaken from the anesthesia spontaneously. The noninstrumented control group did not undergo vaginal dilation.

### 2.7. Suprapubic Tube Implantation

The surgical procedure was carried out under 1.5% isoflurane anesthesia [23]. Two days after VD, an SPT (PE-10 tubing, Clay Adams, Parsippany, NJ, USA) was implanted in the bladder. Key points of the operation were as follows: (1) a midline longitudinal abdominal incision was made, 0.5 cm above the urethral meatus; (2) a small incision was made in the bladder wall, and PE-10 tubing with a flared tip was implanted in the bladder dome; and (3) a purse-string suture with 8-0 silk was tightened around the catheter, which was tunneled subcutaneously to the neck, where it exited the skin.

### 2.8. LPP and MUCP Measurement

Two days after implanting the bladder catheter, the LPP was assessed in these mice under urethane anesthesia. The bladder catheter was connected to both a syringe pump and a pressure transducer. Pressure and force transducer signals were amplified and digitized for computer data collection at 10 samples/second (PowerLabs, AD Instruments, Bella Vista, Australia). The mice were placed supine at the level of zero pressure while bladders were filled with room temperature saline at 1 mL/h through the bladder catheter. If a mouse voided, the bladder was emptied manually using Crede's maneuver. The average bladder capacity of each mouse was determined after 3–5 voiding cycles. Subsequently, the LPP and MUCP were measured in the following manner [23, 24]. When half-bladder capacity was reached, gentle pressure with one finger was applied to the mouse's abdomen. Pressure was gently increased until urine leaked, at which time the externally applied pressure was quickly removed. The peak bladder pressure was taken as the LPP. At least three data were obtained for each animal, and the mean LPP was calculated.

Urethral pressure profile (UPP) was assessed in these mice under urethane (1 g/kg, i.p.) anesthesia. The bladder catheter (PE-10 tubing, Clay Adams, Parsippany, NJ, USA) was connected to a syringe pump with room temperature saline at 1 mL/h. The urethral catheter was connected to a pressure transducer. A withdrawal speed of 10 *μ*m per minute was used. Pressure and force transducer signals were amplified and digitized for computer data collection at 10 samples/second (PowerLabs, AD Instruments, Bella Vista, Australia). Three successive profiles were obtained in the supine position. The urethral closure pressure (Pclose) is the difference between the urethral pressure (Pure) and the bladder pressure (Pves): Pclose = Pure − Pves [25]. Maximum urethral pressure (MUP) and MUCP were determined from the UPP measurements taken.

### 2.9. Western Blot Analysis

Cell extract or urethral tissue was prepared by homogenization of cells in a lysis buffer containing 1% IGEPAL CA-630, 0.5% sodium deoxycholate, 0.1% sodium dodecyl sulfate, aprotinin (10 mg/mL), leupeptin (10 mg/mL), and phosphate-buffered saline (PBS). Cell lysates containing 100 *μ*g of protein were subjected to sodium dodecyl sulfate polyacrylamide gel electrophoresis and then transferred to a polyvinylidene fluoride membrane (Millipore Corp, Bedford, MA, USA). The membrane was stained with Ponceau S to verify the integrity of the transferred proteins and to monitor the unbiased transfer of all protein samples. Detection of myosin, inducible nitric oxide synthase (iNOS), survival motor neuron (SMN) protein, *α*-adrenergic receptor 1a (AdR1a), extracellular superoxide dismutase (SOD3), and glyceraldehyde 3-phosphate dehydrogenase (GAPDH) on the membranes was performed with an electrochemiluminescence kit (Amersham Life Sciences Inc., Arlington Heights, IL, USA) with the use of the antibody derived from rabbit (anti-myosin antibody, 1 : 200 dilution, Millipore, MA, USA; anti-iNOS antibody, 1 : 200 dilution, Abcam, Cambridge, UK; anti-AdR1a antibody, 1 : 200 dilution, Abcam, Cambridge, UK; and anti-GAPDH antibody, 1 : 3000 dilution, Abcam, Cambridge, UK). The intensity of each band was quantified using a densitometer (Molecular Dynamics, Sunnyvale, CA, USA) [26, 27].

### 2.10. Statistical Analysis

The data are presented as mean ± standard deviation (SD) for each group. Statistical differences among groups were determined by one-way analysis of variance (ANOVA) followed by Fisher's LSD as a* post hoc* test. All statistical tests were two-sided. A *P* value less than 0.05 was considered statistically significant. All calculations were performed using the Statistical Package for Social Sciences (SPSS for Windows, release 8.0, SPSS Inc., Chicago, IL, USA).

## 3. Results

### 3.1. Organ Bath Study

**Figure 1:**
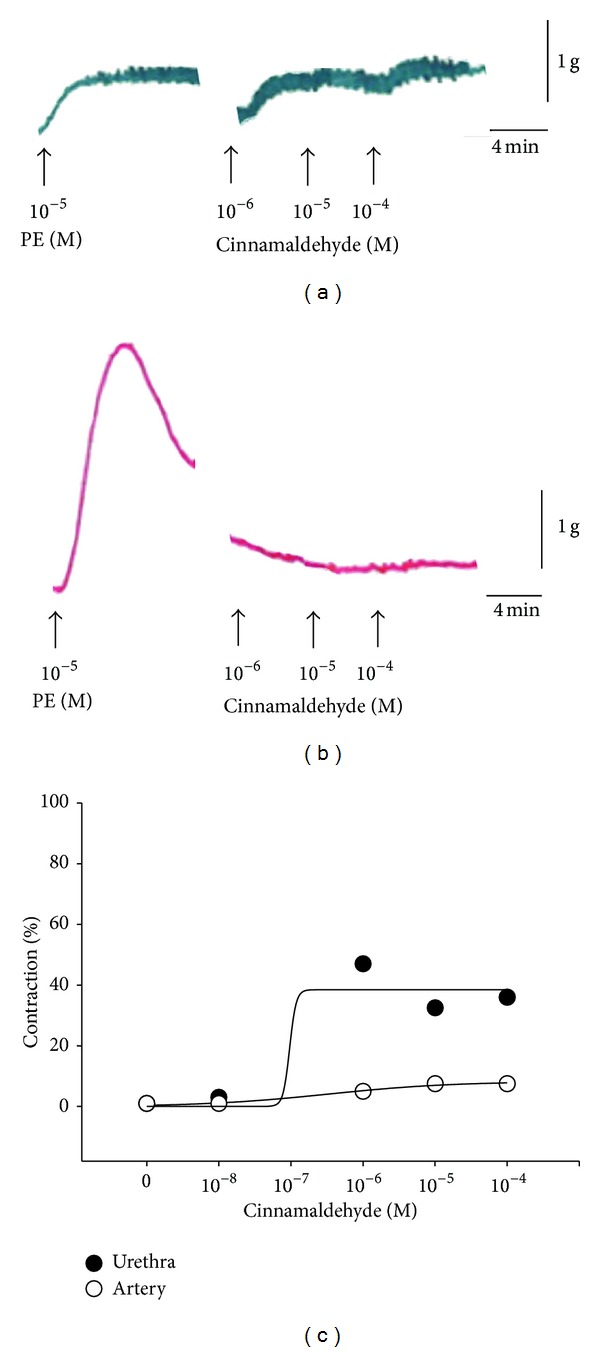
Urethra and artery contraction in organ bath experiment. Representative original traces of the contractile responses to PE and cinnamaldehyde in female pig urethra (a) and artery (b). Contractions induced by 10^−5^ M of PE in the control media were taken as 100%. (c) Graphic representation of concentration-response curves.

Some herbal medicines may have a sympathomimetic effect [28]. The organ bath experiment is the gold standard for functional evaluation of contractile tissues* ex vivo*, such as the urinary tract (including ureters, the urinary bladder, and the urethra) and blood vessels [29, 30]. Figures 1(a) and 1(b) show that the *α*-adrenergic agonist PE has high contractile force of both urethra and artery in the organ bath experiment.* Ramulus Cinnamomi* was found to have the highest efficacy for stimulation of urethral contraction. Therefore, we tested the effect of the major constituent of the* Ramulus Cinnamomi*, cinnamaldehyde, and found that cinnamaldehyde has a stimulating effect on urethral contraction but less effect on renal artery contraction (Figures 1(a)–1(c)).

### 3.2. Effect of Cinnamaldehyde on NO Production and Protein Expressions in the Cell Culture Model

**Figure 2:**
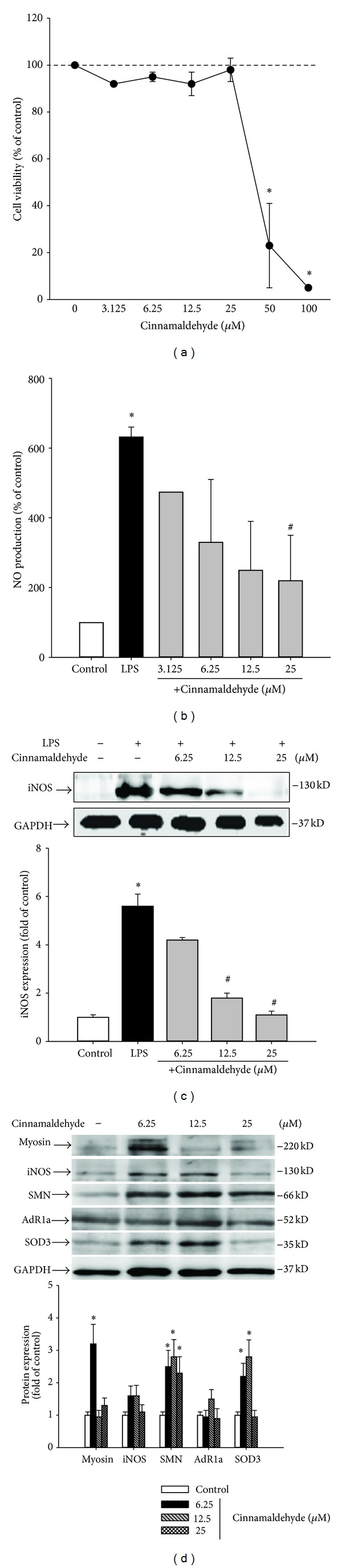
(a) Raw 264.7 cell viability after culture with cinnamaldehyde for 24 h as determined by MTT assay. (b) Effects of cinnamaldehyde on LPS-induced NO production of RAW 264.7 macrophages. Cells were incubated for 24 h with 500 ng/mL of LPS in the absence or presence of cinnamaldehyde. Nitrite concentration in the medium was determined using Griess reagent. (c) Inhibition of iNOS protein expression by cinnamaldehyde in LPS-stimulated RAW 264.7 cells. (d) Modulation of myosin, iNOS, SMN, AdR1a, and SOD3 protein expressions in cinnamaldehyde-treated HBdSMCs. GADPH was used as a loading control. The calculated data were presented as mean ± SD for at least three different experiments. *P < 0.05, compared to control group. ^#^ *P* < 0.05, compared to LPS group.

The primary etiological factor of SUI is vaginal delivery [31], which may provide ischemic [32] and inflammatory damage to the urogenital tract [33]. Ischemia and inflammation cause lower urinary tract dysfunction and activate iNOS expression, thereby increasing NO synthesis and resulting in urethral relaxation [4, 21]. Thus, we tested the effect of cinnamaldehyde on the NO production* in vitro *and iNOS expression in the mouse leukemic monocyte macrophage cell line Raw 264.7. The effect of cinnamaldehyde on RAW 264.7 cell viability was first determined by an MTT assay. Cells cultured with cinnamaldehyde at the concentrations 0, 6.25, 12.5, and 25 *μ*M for 24 h did not change cell viability; however, cinnamaldehyde significantly reduced cell viability at high concentrations (50 and 100 *μ*M) (Figure 2(a)). Therefore, the effect of cinnamaldehyde (≤25 *μ*M) on LPS-induced NO production in RAW 264.7 macrophages was investigated. Nitrite accumulated in the culture medium was estimated by the Griess reaction as an index for NO release from the cells. After treatment with LPS (500 ng/mL) for 24 h, the nitrite concentration increased in the medium. When RAW 264.7 macrophages were treated with different concentrations of cinnamaldehyde together with LPS for 24 h, the cinnamaldehyde dose-dependently reduced nitrite production (Figure 2(b)). In parallel, cinnamaldehyde dose-dependently inhibited LPS-induced iNOS expression (Figure 2(c)).

Our preliminary data of proteomic analysis related to SUI following VD found 68 differentially expressed proteins of the urethra (unpublished data). Majority of the VD-modulated proteins were involved in muscle contraction, metabolites and energy, oxidative stress, regulation of apoptosis, and glycolysis. Since human urethral smooth muscle cells are not commercially available, primary human bladder smooth muscle cells (HBdSMC) were used as a urethral cell culture model to explore the effects of cinnamaldehyde on the expression of several candidate proteins that related to SUI.  Figure 2(d) shows that low-dose (6.25 and/or 12.5 *μ*M) cinnamaldehyde significantly induced myosin, SMN, and SOD3 expressions.

### 3.3. Treatment of SUI by Cinnamaldehyde in the Mice VD Model

**Figure 3:**
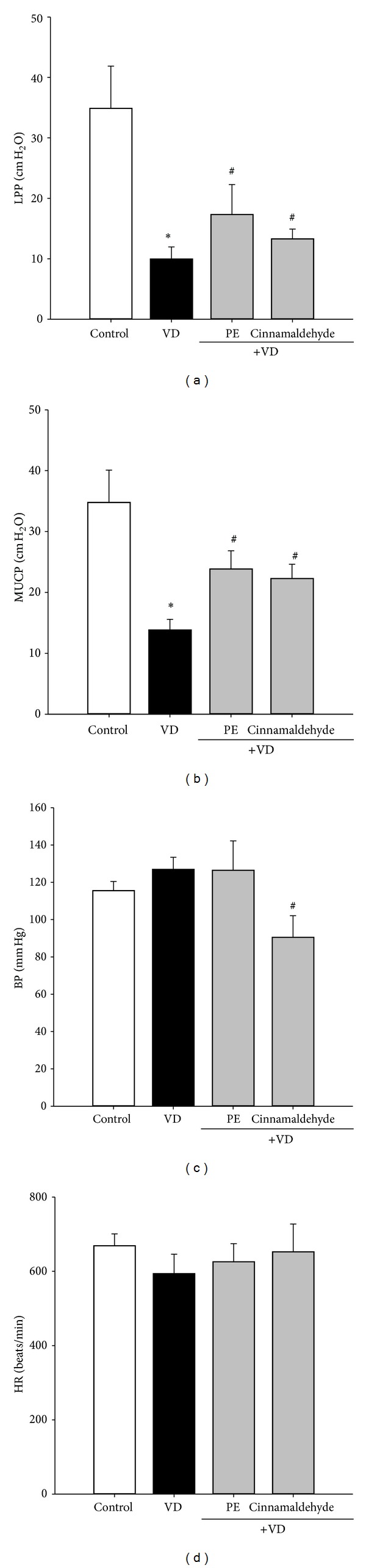
(a) LPP, (b) MUCP, (c) BP, and (d) HR values on the sixth day after VD in the different groups. Each bar represents the mean ± SD of five individual mice. *P < 0.05, compared to control group. ^#^ *P* < 0.05, compared to VD group.

VD has been used for creation of SUI in rats, as evidenced by LPP and MUCP on urodynamic testing [20, 21]. Figures 3(a) and 3(b) show that LPP and MUCP, respectively, were significantly decreased in the VD group as compared to the uninstrumented control group; treatment with both PE and cinnamaldehyde significantly reversed the SUI physical signs. However, as anticipated, BP was slightly increased in the PE group, although this did not reach statistical significance; cinnamaldehyde treatment significantly decreased the BP as compared with the results of both control and VD groups (Figure 3(c)). No significant change was found in HR amount in all experimental groups (Figure 3(d)). The results provide evidence that cinnamaldehyde induces high contractile force of the urethra and low contractile force of blood vessels in the animal models. Cinnamaldehyde may offer therapeutic potential against SUI without the possible side effect of hypertension.

### 3.4. Protein Expressions in the SUI Mice Model

**Figure 4:**
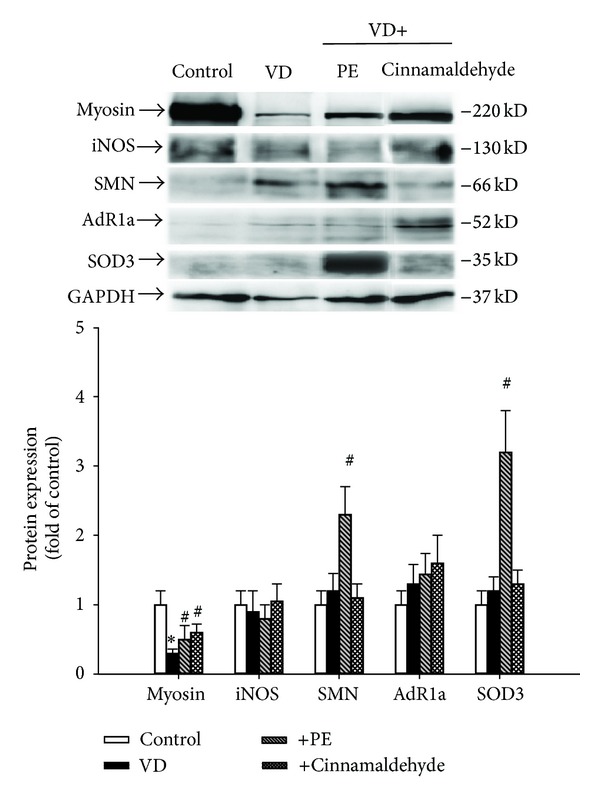
Myosin, iNOS, SMN, AdR1a, and SOD3 expressions on the sixth day after VD in the different groups. Each bar represents the mean ± SD of five individual mice. *P < 0.05, compared to control group. ^#^ *P* < 0.05, compared to VD group.

We used Western blot analysis to confirm several candidate proteins from preliminary data of proteomic analysis related to SUI following VD and tried to explore the possible* in vivo* mechanisms of cinnamaldehyde on SUI.  Figure 4 shows that myosin expression in the urethra was significantly decreased in the VD group as compared with that in the control group; PE and cinnamaldehyde treatments significantly reversed myosin downregulation. No statistical change was found in iNOS and AdR1a expressions. PE treatment significantly increased the expressions of SMN and SOD3.

## 4. Discussion

In the present study,* ex vivo* organ bath assay revealed that* Ramulus Cinnamomi *extract and its major constituent cinnamaldehyde cause high contractile force of urethra and low contractile force of blood vessels. In the VD-induced SUI model in mice, cinnamaldehyde treatment significantly reversed the SUI physical signs without the side effect of hypertension. The modulation of several SUI-related proteins including myosin, iNOS, SMN, AdR1a, and SOD3 may play some crucial roles in the SUI progression and medical treatment.

Integrative medicine is commonly used for health conditions in middle-aged women, and the traditional Chinese herbal medicines have been used to ameliorate the physical signs of SUI, but evidence regarding the benefits is still lacking.* Ramulus Cinnamomi* is the twig of an evergreen tree belonging to the Lauraceae family. It is commonly used as Chinese medicine for gastritis, dyspepsia, blood circulation disturbances, inflammation, and also incontinence [11]. Its extracts contain several active components such as essential oils including cinnamaldehyde [34]. This study provides the first evidence that cinnamaldehyde increased urethra contraction but had less effect on renal artery contraction, suggesting its potential on SUI treatment. A possible reason is that urethral and arterial smooth muscle cells have very different pharmacological profiles [35], such as the different distribution of adrenoceptors in these tissues. Since one of the more obvious side effects of *α*-agonists is the stimulation of vasomotor responses and BP elevation, suggesting the complementary value of cinnamaldehyde to conventional PE treatment on SUI. Moreover, cinnamaldehyde has pleiotropic pharmacological properties, such as antioxidant, anti-inflammatory, and antiskin aging activities [36], suggesting the possible advantages of cinnamaldehyde over PE in SUI treatment.

The primary etiological factor of SUI is vaginal delivery [31], which may provide ischemic [32] and inflammatory [37] damage to the urogenital tract [33]. Ischemia and inflammation cause lower urinary tract dysfunction [37], activate iNOS expression, and increase NO synthesis resulting in urethral relaxation [4, 21].* In vitro* models such as macrophage or other cell lines are useful materials with a steady high-level production of NO. These results are consistent with previous studies [11, 38, 39] that cinnamaldehyde inhibits LPS-induced iNOS expression and NO production [40]. It also suggests the possible inhibitory effect of cinnamaldehyde on macrophage-mediated inflammatory responses. Interestingly, low-dose cinnamaldehyde appears to slightly enhance iNOS in the HBdSMCs, although this did not reach statistical significance. NOS activation as a result of trauma (calcium influx) leads to NO production, which activates its downstream receptors. This event may lead to one or more systemic effects including dilatation in the cardiovascular, respiratory, and urinary systems [41]. However, NO has dual role as a protective and toxic molecule. This paradoxical dichotomy is leaving investigators with a double challenge to determine the net impact of NO [42]. Cinnamaldehyde has been shown to induce endothelium-dependent and -independent vasorelaxant action [43, 44]. The other molecular mechanisms by which cinnamaldehyde inhibits urethral contraction need to be further elucidated. A comprehensive and dynamic view of the cascade of molecular and cellular events underlying urinary biology will allow investigators to exploit the potential properties of NO.

Myosin molecules consist of two heavy chains. Myosin heavy chains play a crucial role in modulating smooth muscle contraction [45]. In an animal model, increasing load induces significant smooth muscle hypertrophy, which is associated with a downregulation of myosin heavy chain expression. This contributes to the decreased smooth muscle contractility [46]. In this study, the protein expression of myosin in both HBdSMCs and mice urethral tissues was examined. The results indicated that low-dose cinnamaldehyde enhanced smooth muscle myosin protein levels. In the VD mice, myosin expression in the urethra was significantly decreased, and cinnamaldehyde treatment could markedly reverse the myosin downregulation. It is possible that VD in the urethra induces dysfunction of the smooth muscle cells, which alters contractility, leading to altered urethral performance and decreased compliance. These data suggest the potential pharmacologic effect of cinnamaldehyde on smooth muscle contractile properties with their myosin expression.

SMN protein is necessary for the assembly of Sm proteins onto the small nuclear RNAs to form small nuclear ribonucleoproteins (snRNPs) [47]. The mutations of SMN gene result in spinal muscular atrophy, a common neurodegenerative disease. However, the viability and sensitivity to stresses of other cell types also need to be determined. Liu et al. established HeLa stable cell line with inducible SMN knockdown to study its viability and sensitivity to oxidative stress. Their results suggested that the there was only a slight decrease in the proliferative rate of SMN knockdown cells. In contrast, after H_2_O_2_ reached certain concentrations, the survival rate of SMN knockdown cells decreased significantly. Their data indicate that SMN knockdown alone is not critical to cell viability. However, when SMN knockdown cells are under stress, such as oxidative stress [48], their survival rate may significantly decrease [47]. In parallel, SOD3 (extracellular superoxide dismutase, EC-SOD) was shown to be the predominant form in extracellular fluids. Many studies have focused on the antioxidative effect of SOD3 in the lung and vascular wall where it is highly expressed. Thus, the roles of SOD3 as an antioxidative enzyme in ROS-mediated ischemia and inflammation have been extensively studied. However, Kwon et al. revealed that the role of SOD3 in inflammation is not simple because of radical scavenging; it affects immune responses and signal initiation [49] as VD may provide ischemic and oxidative damage to the urogenital tract [33]. This data showed that cinnamaldehyde and PE induced SMN and SOD3 in HBdSMCs and urethral tissues of mice, respectively, suggesting that SMN and SOD3 may play important roles in pharmacological prevention of SUI.

Traditional *α*-adrenergic stimulating drugs cause urethral contraction and have unwanted side effects including vasomotor stimulation, restlessness, and insomnia [4, 12]. Sympathetically mediated urethral tone is essential for the maintenance of continence and involves the activation of postjunctional *α*_1_-adrenoceptors. The contraction of urethral circular smooth muscle is mediated via *α*_1_-adrenoceptors with the pharmacological characteristics [50]. Yono et al. showed that doxazosin (*α*-blocker) treatment caused an upregulation in the mRNA levels of *α*_1_-adrenoceptors in the rat urethra, indicating that chronic doxazosin treatment may cause an alteration in the properties of *α*_1_-adrenoceptors [51]. These results showed that cinnamaldehyde did not significantly modify the AdR1a expression, suggesting that alteration in the properties of *α*_1_-adrenoceptors is probably not involved in cinnamaldehyde pharmacology.

## 5. Conclusion

The major constituent of the Chinese medicinal herb* Ramulus Cinnamomi*, cinnamaldehyde, significantly reversed the VD-induced SUI physical signs without the side effect of hypertension. The modulation of several SUI-related proteins including myosin, iNOS, SMN, and SOD3 may play some crucial roles in the therapeutic approach against SUI. The exact mechanism underlying its effects remains unclear, and further experimental and clinical studies are required to elucidate the mechanism(s) responsible for its pharmacological activities.
